# Different changes of microarchitectures of cortical and cancellous bones in sheep femoral head after long-term glucocorticoid interventions

**DOI:** 10.1038/s41598-018-28433-7

**Published:** 2018-07-03

**Authors:** Yuan-Hui Li, Fu-Qiang Gao, Li-Ming Cheng, Mian-Dong Zeng, Qin-Ye Qiu, Ming Ding

**Affiliations:** 1 Department of Orthopaedics, The Third Affiliated Hospital of Guangzhou Medical University, Guangzhou, 510150 China; 2 Department of Orthopaedics, China-Japan Friendship Hospital, Beijing, 100029 China; 3 Orthopaedic Research Laboratory, Department of Orthopaedics O, Odense University Hospital, Institute of Clinical Research, University of Southern Denmark, Odense C, Denmark

## Abstract

This study investigatedthe different effects of long-term glucocorticoid (GC) interventions on the microarchitectures of cortical and cancellous bones of the femoral head. Eighteen female skeletal mature sheep were randomly allocated into 3 groups, 6 each. Group 1 received prednisolone interventions (0.60 mg/kg/day, 5 times weekly) for 7 months. Group 2 received the same interventions as Group 1 and then further observed 3 months without interventions. Control Group was left nonintervention. After killing the animals, all femoral heads were scanned by micro-CT to determine their microstructural properties. In cancellous bone of femoral head, GC interventions led to significant decrease of bone volume fraction, trabecular thickness, trabecular separation, but increase of structure model index and bone surface density (p < 0.05). While in cortical bone, there were no differences between the Group 1 and in microstructural properties (p > 0.05) except greater trabecular thickness in the control group. In addition, three months after cessation of glucocorticoid interventions, most microstructural properties of cancellous bone were significant reversed, but not cortical thickness of femoral head. In contrast to cancellous bone, the microarchitectures of cortical bone were not changed obviously after long-term GC interventions.

## Introduction

Many experiments and clinical studies have confirmed the causal relationship between the hormone and the necrosis of the femoral head^1–4^. With the widespread use of glucocorticoid (GC) in clinic, the number of cases of femoral head necrosis caused by glucocorticoid is increasing, that has attracted more and more attention. GC induced avascular necrosis of femoral head (ANFH) is characterized by multiple lesions, high risk of bilateral hip joints involvement, and poor effectiveness of conservative treatment. Eventually hip dysfunction leads to poor quality of life. Unfortunately, the affected population is mostly young or middle-aged people^5^. The integrity and mechanical strength of the cortical bone of femoral head are the key factors for the occurrence and development of femoral head necrosis. The mechanism of GC induced necrosis of the femoral head is not very clear^6^, the destruction of the cortical bone and the consequent collapse of femoral head are the turning points in the process of femoral head necrosis (Supplementary file).

The inner of the femoral head is composed of a large number of trabecular bone, which can effectively absorb and dissolve the impact load and shock energy. In the event of the structure of trabecular bone changes, the mechanical strength of trabecular bone will altered^7,8^. Research has reported that mechanical properties of trabecular bone in ANFH decreased significantly. The decreased properties of cortical bone have very close relation with collapse of femoral head and it was regarded as the direct cause of the collapse of the femoral head^9^. This was confirmed in our previous micro-CT study. The cortical bone of the femoral head is an important structure for cartilage adhesion and support. Research has shown that subchondral bone of femoral head could decrease 30% of the total load through hip joint^10^. Additionally, subchondral cortical bone can transfer the load from the local contact surface to trabecular bone around^11^. Given the above, the cortical bone of the femoral head plays important roles in load bearing, cartilage support and stress dispersion of femoral head. It is important to study GC-induced microarchitectural changes of cortical and cancellous bone. The results will be benefit for further research of the occurrence and development of the GC-induced femoral head necrosis.

The size and anatomy of sheep skeleton, and bone remodeling activity in sheep are similar to those of human bone^12,13^. Sheep have been widely used as an experimental model of skeleton disease^8,12^. There are many studies on the effects of GC on the microarchitecture and mechanical strength of cancellous bone of femoral head, but few about the cortical bone of femoral head. Microstructural study of cortical bone of femoral head induced by glucocorticoid and research on GC-induced microstructural differences between cortical and cancellous bone of femoral head help to understand the occurrence and development of femoral head necrosis, but also help to determine prognosis and treatment choice.

The aims of this study, mimicking clinical situation, were to explore effects of long-term GC interventions on microstructural changes of cortical and cancellous bone of femoral head. We hypothesized that significant microstructural changes would occur after long-term GC interventions, and the microarchitecture would be reversed after timely cessation of glucocorticoid.

## Results

The eighteen female skeletal mature Merino sheep were randomly allocated into 3 groups, 6 sheep each. The mean body weight at the beginning of the experiment was 55 ± 10 kg (range, 36–69 kg) and did not differ between the 3 groups. These sheep had been used previously for investigating the effects of GC on the microstructural properties of cancellous bones from the vertebra, femurs, and tibias. This study focused on the effects of GC on femoral head in sheep^7^. Group 1 (GC-1) received GC interventions by subcutaneous injection, i.e., prednisolone 0.60 mg/kg/day, 5 times weekly (10 mg/mL prednisolone acetate, Prednisolone acetate Vet., Intervet, DK-2740 Skovlunde, Denmark) for 7 months. Group 2 (GC-2) received the same interventions as GC-1 for 7 months followed by 3 month without interventions in order to further observe if effects of GC on bone was maintained. The total observation time was 10 months. During the last 2 weeks of Prednisolone interventions, the dose was halved weekly, resulting in a dose of 0.30 mg/kg/day and 0.15 mg/kg/day, respectively. After this, the prednisolone interventions was discontinued. By this procedure, the dosage was gradually decreased to reduce symptoms associated with GC abatement described as Addison’s disease. Group 3 was served as the control group, and the sheep were left without interventions for 7 months.

### Physical activities of the sheep

Generally, all sheep had normal daily activities, and the body weights did not differ between the groups (ANOVA, P = 0.66–0.85). Three of 6 treated sheep in GC-1 and 4 of 6 treated sheep in GC-2 had lost their hairs toward the end of the interventions indicating a skin reaction. Otherwise, the sheep tolerated GC interventions well and did not show any other side effects during the 7 months of interventions.

### Cancellous bone microarchitecture

**Figure 1:**
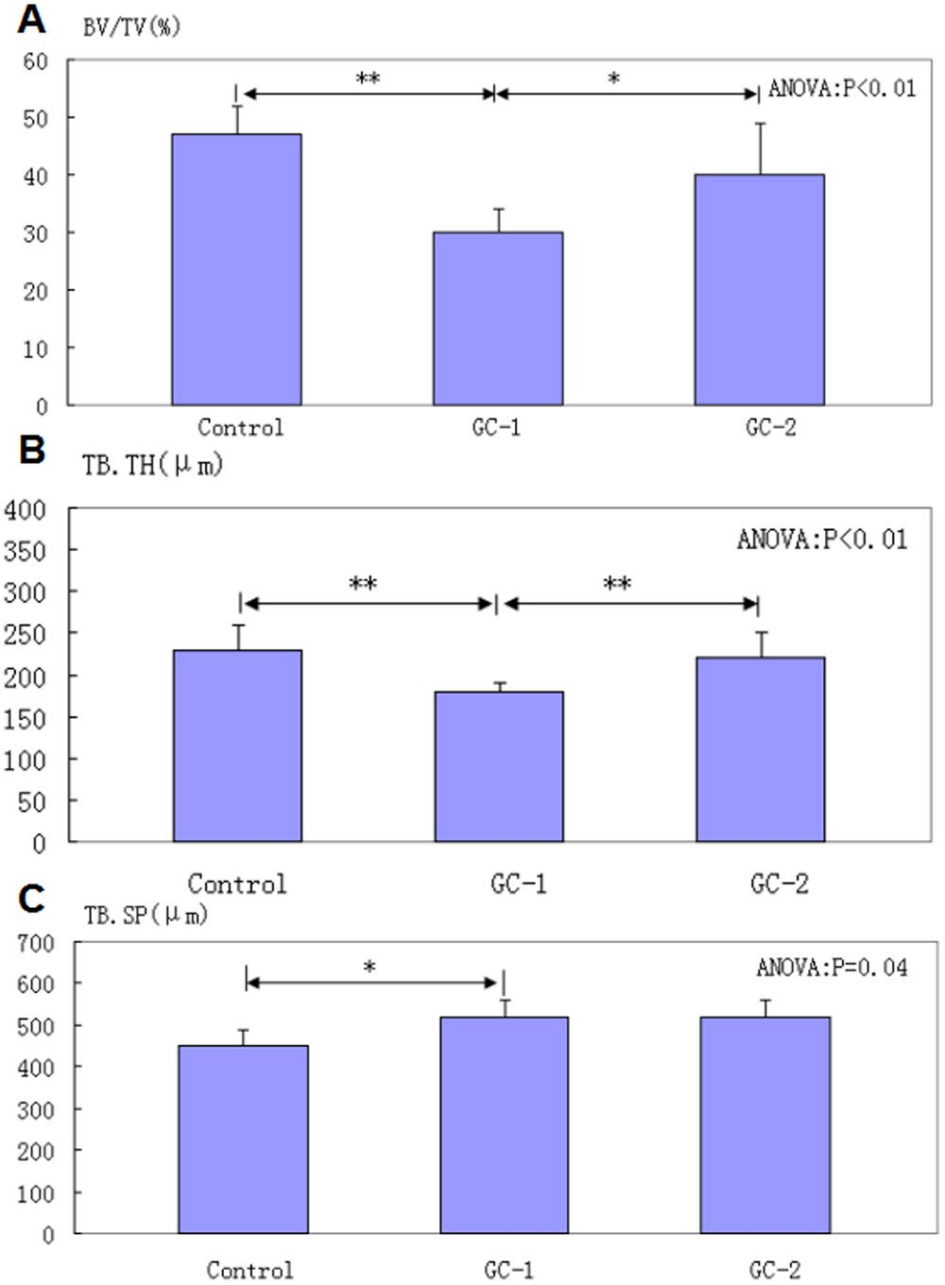
Microarchitectural properties of cancellous bone in femoral head. (**A**–**C**) BV/TV, TB.TH and TB.SP of cancellous bone in different groups. *P < 0.05, **P < 0.01. BV/TV, bone volume fraction; TB.TH, trabecular thickness; TB.SP, trabecular separation.

For cancellous bone, significant microstructural changes were observed in the GC-1 compared with the control group,except the appearance of micro-CT 3D images. There were significant decrease in bone volume fraction (BV/TV) (P < 0.01), trabecular thickness (TB.TH) (P < 0.01) and trabecular separation (TB.SP) (P < 0.05). In addition, TB.SP, Bone surface density (BS/TV) and Structure model index (SMI), which reflects plate-like and rod-like trabecular bone, were increased in the GC-1 compared to the control group (P < 0.05). There was no marked changes in connectivity density (Conn.D), BS/TV and trabecular number (TB.N) between the GC-1 and the control group (P > 0.05). Three months after cessation of GC interventions, the microarchitecture of trabecular bone in the GC-2, compared with GC-1, revealed significant increase in BV/TV(P < 0.05), TB.TH(P < 0.01), and decrease in SMI (P < 0.05), and BS/TV(P < 0.05). There were no significant difference between GC-1 and GC-2 in TB.SP, BS/TV and TB.N (Table 1 and Fig. 1).

**Table 1:** Three-Dimensional Microarchitectural Properties of Cancellous Bone in femoral head.

Groups	SMI (−)	Conn.D (1/mm³)	BS/TV (1/mm)	BS/BV (mm)	TB.SP (μm)	TB.TH (μm)	TB.N (1/mm)	BV/TV (%)
Control	−1.51 ± 1.05	5.94 ± 5.23	4.26 ± 0.49	8.27 ± 1.87	450 ± 40	230 ± 30	2.07 ± 0.56	47 ± 5
GC-1	0.44 ± 0.5	7.68 ± 1.3	4.0 ± 0.39	13.52 ± 0.67	520 ± 40	180 ± 10	1.84 ± 0.13	30 ± 4
GC-2	−1.02 ± 1.20	4.11 ± 1.59	3.86 ± 0.28	10.16 ± 2.25	520 ± 40	220 ± 30	1.73 ± 0.09	40 ± 9
ANOVA (P)	P < 0.01 GC-1 > GC-2, Control	P = 0.20	P = 0.26	P < 0.01 GC-1 > GC-2 > Control	P = 0.04 Control < GC-1	P < 0.01 GC-1 < GC-2,Control	P = 0.25	P < 0.01 GC-1 < GC-2 < Control

GC, Glucocorticoid; SMI, Structure model index; Conn.D, Connectivity density; BS/TV, Bone surface density; BS/BV, surface to volume ratio; TB.SP, trabecular separation; TB.TH, trabecular thickness; TB.N, trabecular number; BV/TV, bone volume fraction; ANOVA, Analysis of variance.

### Cortical bone microarchitecture

**Figure 2:**
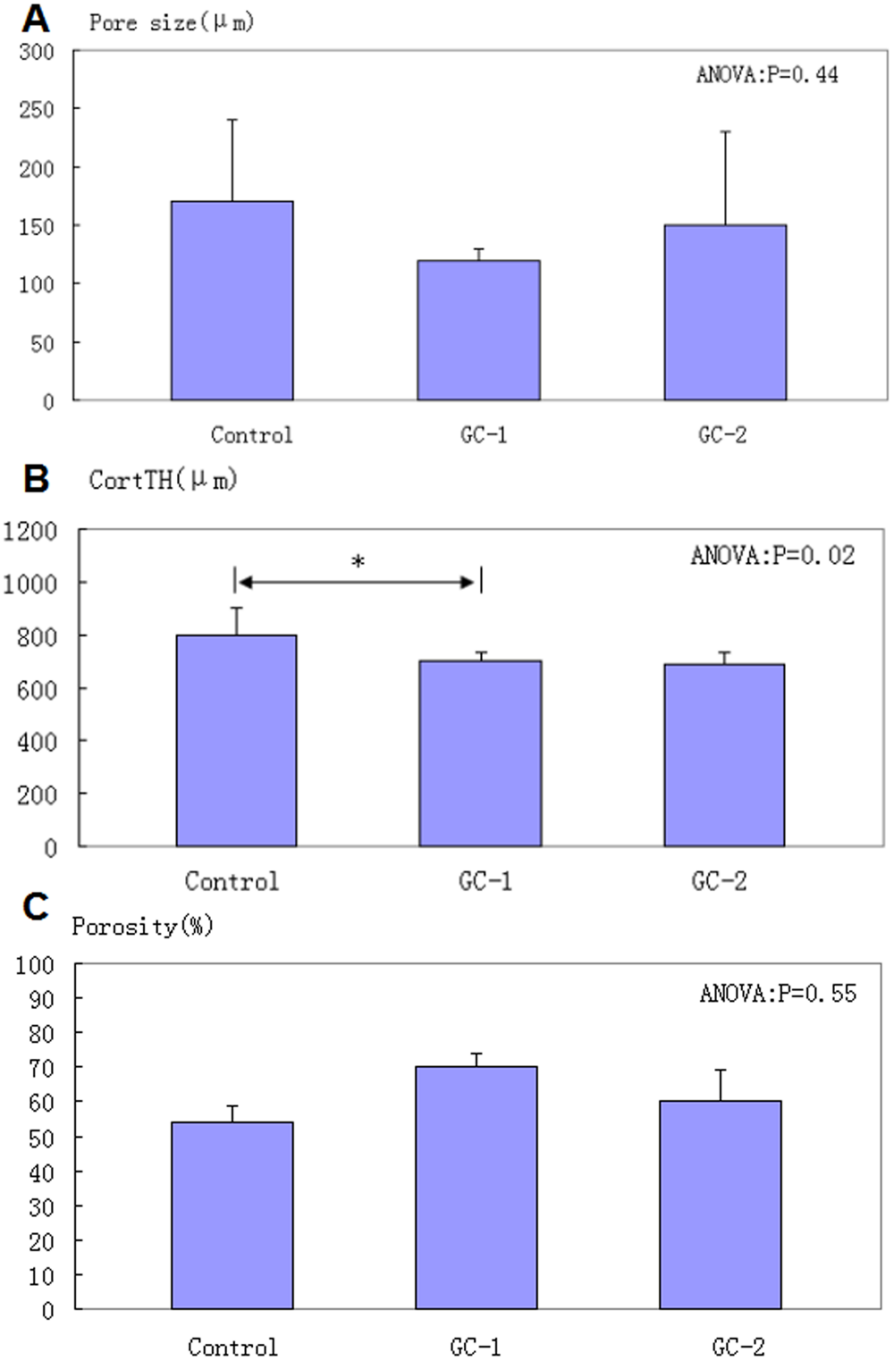
Microarchitectural properties of cortical bone in femoral head. (**A**–**C**) pore size, CortTH and porosity of cortical bone in different groups. *P < 0.05, **P < 0.01. CortTH, Cortical thickness.

Different from cancellous bone microarchitecture, porosity, and pore size did not show significant changes between the GC-1and the control group in cortical bone. A similar result of these microarchitectural characteristics in cortical bone was also seen in the GC-2 compared with the GC-1. And differences in the appearance of micro-CT 3D images was not noticeable in the three groups. However, cortical thickness was thinner in the GC-1 than the control group (P < 0.05). After cessation of glucocorticoid 3 months, there was no significant difference in cortical thickness between GC-1 and GC-2 (Table 2 and Fig. 2).

**Table 2:** Three-Dimensional Microarchitectural Properties of Cortical Bone in femoural head.

Groups	BS/TV (1/mm)	BS/BV (mm)	Pore size (μm)	CortTH (μm)	Porosity (%)
Control	1.93 ± 0.45	1.69 ± 0.5	170 ± 70	800 ± 100	54 ± 4.58
GC-1	1.78 ± 0.22	1.82 ± 0.55	120 ± 10	700 ± 30	70.33 ± 3.80
GC-2	1.98 ± 0.38	1.88 ± 0.57	150 ± 80	690 ± 40	60.38 ± 8.64
ANOVA (P)	P = 0.63	P = 0.75	P = 0.44	P = 0.02 GC-1 < Control	P = 0.55

GC, glucocorticoid; BS/TV, Bone surface density; BS/BV, surface to volume ratio; CortTH, Cortical thickness; ANOVA, Analysis of variance.

## Discussion

The interaction of cortical bone and cancellous bone in the femoral head plays important roles in maintaining the shape of the femoral head and absorbing the load through hip joint. Research indicated that long-term glucocorticoid therapy could lead to the decrease of BV/TV, TB.TH and the mechanical strength of trabecular in cancellous bone^7,8,12,14^. This study investigated the changes of cancellous and cortical microarchitecture after long-term GC interventions to seek possible cause of severely disordered cancellous microarchitecture that did not lead to ANFH and collapse of femoral head. Osteoporotic microarchitectural changes were induced by GC in lumbar vertebra and proximal tibia in GC-1, after receiving GC interventions by subcutaneous injection prednisolone 0.60 mg/kg/day, 5 times weekly for 7 months^7^. Additionally, the GC treated sheep had normal activities, although many of them had lost hairs, indicating skin reaction to GC interventions. The loss of hair might be an early indication of immunosuppressive effect after GC interventions. The body weight of the sheep did not differ between groups, while treated animal had relative lower bone density. This condition is similar to the patients received long-time glucocorticoid interventions.

According to the glucocorticoid dose in this study, the GC groups received the prednisolone interventions up to 84 mg/KG. This dose is larger than that cause the necrosis of the femoral head in some animal experimental and clinical studies^15–19^. In light of a clinical study^19^, if patients were treated with total dosage of prednisolone over 2000 mg, the rate of bone necrosis was as high as 45.6%. As such, if an adult (weight, 50 kg) had been treated with the prednisolone dose as used in sheep in this study (84 mg/KG), he would have received the total dose of 4200 mg, which is much more than 2000 mg. Therefore, this prednisolone dose (84 mg/KG) would have been sufficient to induce osteonecrosis of femoral head. It was benefit for study different effects of glucocorticoid on microarchitectures of cortical and cancellous bones before osteonecrosis of femoral head.

Recent studies have demonstrated that microstructural changes of bone would significantly affect its mechanical properties^7,12^. In the present study, GC-induced microarchitectural changes in cancellous bone of the femoral head were observed in GC-1 compared with control group. BV/TV and TB.TH declined by 37.2% and 21.8% respectively after 7 months of GC interventions. Additionally, TB.SP and SMI revealed a significantly increase in GC-1 when compared with control group. These data indicated that significant microarchitectural changes in cancellous bone of femoral head with lower bone density, thinner trabeculae, decreased plate-like trabeculae, and great trabecular separation. SMI was used to evaluate the structural morphology of trabecular bone. Trabecular bone structure degeneration increased when SMI value enlarged. In this study, SMI value increased from control group to GC-1, which implied trabecular bone structure degenerated obviously after long-term GC interventions. These microstructural changes of cancellous bone in femoral head would weaken the mechanical properties of the trabecular bone^7,12^. In this animal experiment, other than cortical thickness (CortTH) was thinner by 12.3%, other 3-D microarchitectural properties of cortical bone in GC-1, such as porosity, pore size, bone surface density and surface to volume ratio had no significant difference compared to the control group. These results indicated that the microarchitecture of cortical bone of femoral head did not undergo significant changes after 7 months of GC interventions. The changes in cortical and cancellous bones of femoral head were different after long-term GC interventions. Although significant changes occurred in cancellous bone microarchitecture, only slight changes in cortical bone were observed. Throughout this experiment, we did not found ANFH and collapse of femoral head by micro-CT.

Thus, cancellous bone of femoral head was affected earlier and more severely by GC interventions than in cortical bone. Similar results at other skeletal sites have been documented in humans due to the role of GC^7,8^. Taken together, this phenomenon implying that during the long-term GC interventions, cortical bone might play a key role in maintaining the microarchitecture and mechanical strength of the femoral head and protecting the cancellous bone. In addition, three months after cessation of GC interventions, most microarchitectural properties of cancellous bone were improved significantly. This revealed that the early adverse effect of the GC on the cancellous bone of femoral head was partly reversible after cessation of GC interventions. However, the decreased cortical thickness after 7 months of GC interventions did not ameliorate after cessation of GC suggesting a detrimental cortical bone after GC interventions.

There are several limitations of this animal experiment. Firstly, we did not find ANFH and collapse of femoral head that was only assessed by micro-CT, and no histomorphometry was performed. Secondly, Standard deviations in some indicators, especially SMI in cancellous bone, were slightly big owing to small sample volume and individual variation in sheep. Thirdly, studies have confirmed that the hip anatomy of sheep was similar to those of human^19–21^, but the sheep is a kind of quadrupeds, the biomechanics of hip was different from human^22^. Fourthly, biomechanical test was not done for further mechanical research of trabecular bone.

In conclusion, the present study demonstrated that in the process of long-term glucocorticoid interventions, not like cancellous bone,the microarchitectures of cortical bone were not changed obviously, and this phenomenon implied cortical bone may play key roles in maintaining the shape and mechanical strength of the femoral head and protecting the cancellous bone. The early adverse effect of the GC on the cancellous bone of the femoral head is mostly reversible, but not on cortical bone three months after cessation of glucocorticoid interventions.

## Materials and Methods

### Experimental Animals

Eighteen female sheep (4–6 years of age) were purchased from local farmers at the region of southern Denmark, and included in the experiment. All animals were healthy ex-breeders, and the sheep were acclimated for a period of 2 months prior to the initiation of experiments. During the experiments, the sheep were housed in outdoors paddocks with shelter, and received restricted diet grass pellets, i.e., low calcium and phosphorus intakes (0.55% calcium and 0.35% phosphorus, FAF, DK-5000 Odense C, Denmark) and hay. The daily activities of the sheep were monitored for normality, and at the end of the study, their body weights were recorded. This experimental protocol was in accordance with the Danish Animal Research guidelines, and was approved by the Danish Animal Experiments and Inspectorates.

### Sample preparation

After sacrificed, the femoral heads of sheep were harvested. A diamond-coated band saw (Exakt, Norderstedt, Germany) and a saw microtome 1600 (Ernst Leitz Wetzlar GmbH, Wetzlar, Germany) were used for the sawing procedures at low speed, and water was irrigated continuously during sawing. A saw was made beneath the femoral head to obtain the femoral head samples.

### Microcomputed Tomography (Micro-CT)

The complete femoral head specimens were micro-CT scanned(vivaCT 40, Scanco Medical AG., Brüttisellen, Switzerland) to quantify the 3D microarchitectural properties of trabecular bone and cortical bone. The scanned micro-CT images had 3D reconstruction cubic voxel sizes of 25 × 25 × 25 μm³ (1024 × 1024 pixels). The micro-CT images were segmented using the segmentation techniques described in detail previously with slight modification to obtain accurate 3D imaging datasets. The microarchitectural properties of the cancellous bone and cortical bone were calculated using true, unbiased, and assumption-free 3D methods.

For cancellous bone, bone volume fraction (BV/TV), trabecular number (TB.N) and trabecular thickness (TB.TH) were determined. Structure model index (SMI), a differential analysis of the triangulated bone surface of a structure, and connectivity density (Conn.D), a topological approach were calculated. Bone surface density (BS/TV), surface to volume ratio (BS/BV), and trabecular separation (TB.SP) were also calculated (Fig. 1, Tables 1 and 2). For cortical bone, cortical thickness (CortTH), porosity, pore size, bone surface density, and surface to volume ratio were calculated.

### Statistical analysis

The results were presented as mean ± standard deviation (SD), and were analyzed statistically using statistics software SPSS version 10.0.7 (SPSS Inc. Chicago, IL). Firstly, normality and equal variance of the data were checked. Secondly, one-way analyses of variance (ANOVA) were performed among the 3 groups, and the post hoc multiple comparisons were adjusted using Bonferroni test or Dunnett test as appropriate. A P < 0.05 was considered significant.

## Electronic supplementary material

Supplementary Information
