# Supplementary material for: Different changes of microarchitectures of cortical and cancellous bones in sheep femoral head after long-term glucocorticoid interventions
Source: Sci Rep. 2018 Jul 3;8:9988. doi: 10.1038/s41598-018-28433-7 (PMC6030221; doi:10.1038/s41598-018-28433-7)

Different changes of microarchitectures of cortical and cancellous bones in sheep femoral head after long-term glucocorticoid interventions

Yuan-Hui Li, MD[1*], Fu-Qiang Gao, MD[2*], Li-Ming Cheng, MD[2†*], Mian-Dong Zeng, MD[1*], Qin-Ye Qiu, MD[1*], Ming Ding, MD[3*]

[1] Department of Orthopaedics, The Third Affiliated Hospital of Guangzhou Medical University, Guangzhou, 510150, China

[2] Department of Orthopaedics, China-Japan Friendship Hospital, Beijing 100029,China

[3] Orthopaedic Research Laboratory, Department of Orthopaedics O, Odense University Hospital, Institute of Clinical Research, University of Southern Denmark, Odense C, Denmark

[†] **Corresponding author**:

Li-Ming Cheng, MD Email: zdclm123@126.com

[*] Joint first authors.

These authors contributed epually to this work.

Yuan-Hui Li, MD Email: yuanhui406@126.com

Fu-Qiang Gao, MD Email: gaofuqiang0604@163.com

Li-Ming Cheng, MD Email: zdclm123@126.com

Mian-Dong Zeng, MD Email: 13529611112@139.com

Qin-Ye Qiu, MD Email: qiuqinyedeng@126.com

Ming Ding, MD Email: ming.ding@ouh.regionsyddanmark.dk

Figure 3. Early MR imaging of early-stage osteonecrosis in severe acute respiratory syndrome (SARS) patients with high-dose hormones revealed that hormone-induced osteonecrosis occured in the bone sites-rich in yellow bone marrow (cancellous bone of femoral head and metaphysis of the femur and tibia), while subchondral bone of the femoral head and cortical bone of the long backbones were usually survived.

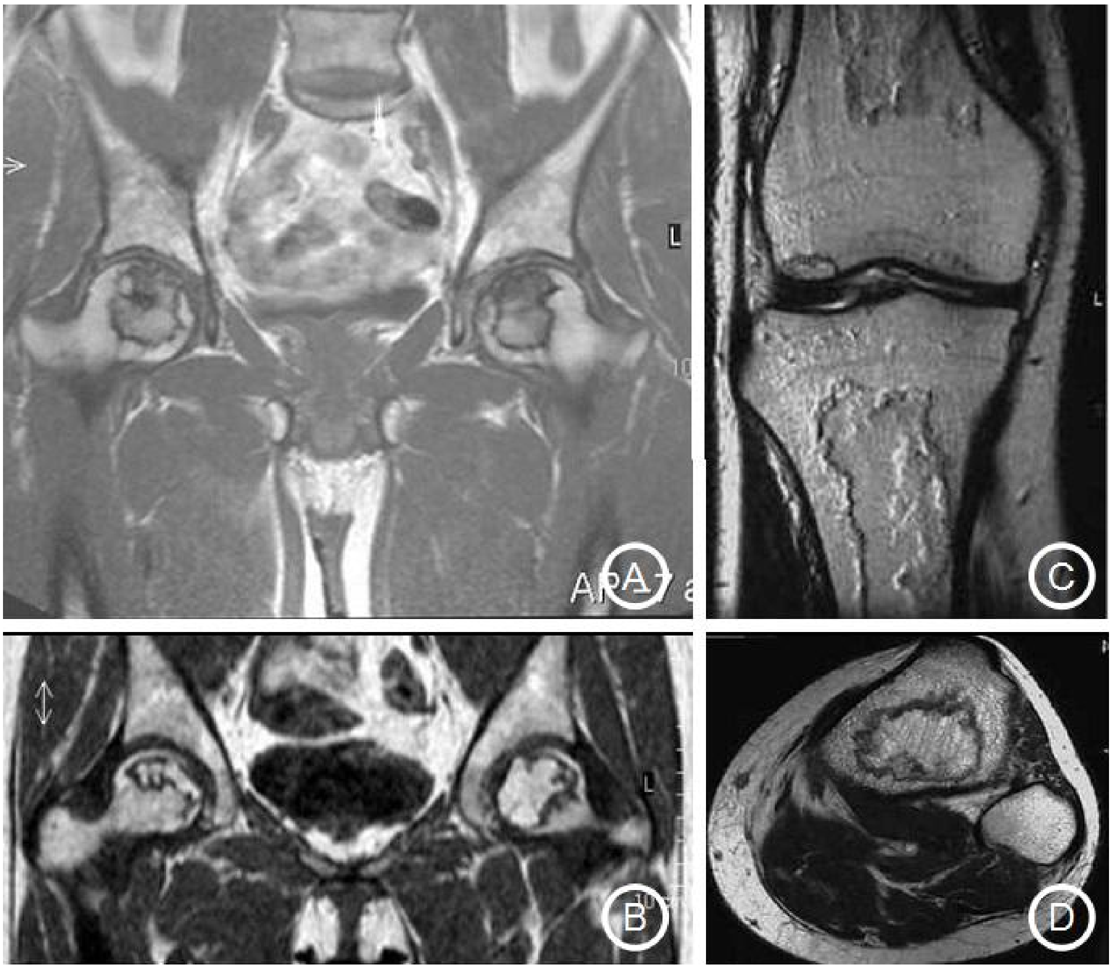

Figure 4. Our previous micro-CT study showed that even in the case of a collapsed necrotic (white arrow head) femoral head, its subchondral bone (cortical bone) remained clearly (white arrow).

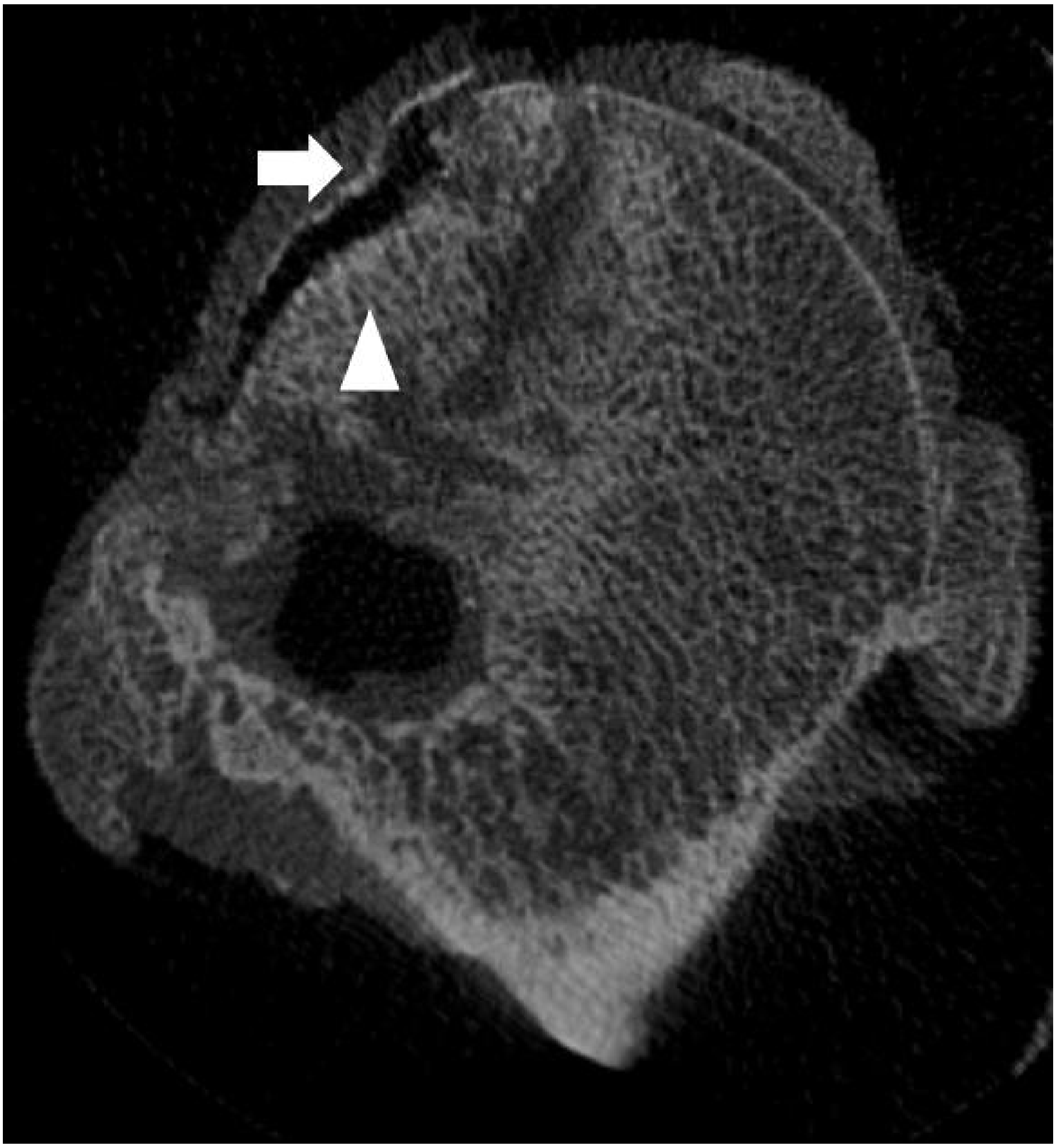

Supplement: Supplementary file 1 — Supplementary Information [file 41598_2018_28433_MOESM1_ESM.pdf]
